# Conditions for the Emergence of Shared Norms in Populations with Incompatible Preferences

**DOI:** 10.1371/journal.pone.0104207

**Published:** 2014-08-28

**Authors:** Dirk Helbing, Wenjian Yu, Karl-Dieter Opp, Heiko Rauhut

**Affiliations:** 1 ETH Zurich – Swiss Federal Institute of Technology Zurich, Chair of Sociology, in particular of Modeling and Simulation, Zurich, Switzerland; 2 Santa Fe Institute, Santa Fe, New Mexico, United States of America; 3 University of Leipzig, Institute of Sociology, Leipzig, Germany; 4 University of Washington, Department of Sociology, Seattle, Washington, United States of America; 5 University of Zurich, Institute of Sociology, Zurich, Switzerland; National Scientific and Technical Research Council (CONICET)., Argentina

## Abstract

Understanding norms is a key challenge in sociology. Nevertheless, there is a lack of dynamical models explaining how one of several possible behaviors is established as a norm and under what conditions. Analysing an agent-based model, we identify interesting parameter dependencies that imply when two behaviors will coexist or when a shared norm will emerge in a heterogeneous society, where different populations have incompatible preferences. Our model highlights the importance of randomness, spatial interactions, non-linear dynamics, and self-organization. It can also explain the emergence of unpopular norms that do not maximize the collective benefit. Furthermore, we compare behavior-based with preference-based punishment and find interesting results concerning hypocritical punishment. Strikingly, pressuring others to perform the same public behavior as oneself is more effective in promoting norms than pressuring others to meet one’s own private preference. Finally, we show that adaptive group pressure exerted by randomly occuring, local majorities may create norms under conditions where different behaviors would normally coexist.

## Introduction

### Research Question

Social norms are among the most important factors that shape social life. Accordingly, a vast literature addresses the emergence and effects of social norms. In the work of classical sociologists, norms play a pivotal role in explaining various aspects of society. For example, in Talcott Parsons’ action frame of reference, a “normative orientation” and “the mutual interlocking of expectations and sanction” is “rooted … in the deepest fundamentals of the action frame of reference” [1]. Émile Durkheim’s theory of morality implies detailed propositions about the extent to which “the division of labor is linked with our whole moral life” [2]. In contemporary sociology, norms are still an important topic [3]–[5]. Norms are also the subject of cultural anthropology, which includes the study of norms as aspects of culture [6], of economics [7]–[8], of game theory [9]–[10], of jurisprudence [11]–[12], and of political science [13].

Despite this extensive literature, our knowledge about the emergence of social norms is still incomplete. While basic variables that cause the emergence of norms have been identified in the past (see the Theory section below), we need to learn more about *dynamic processes* that lead to the emergence of new norms. For example, it is known that externalities are an important factor of norm emergence. However, the exact processes leading to the emergence of norms and normative change under different conditions and the exact preconditions for this process are not clear. An important question is thus how to explain the *dynamics of norm emergence*, not in the sense of the establishment of a norm by overcoming defection, but in the sense of the *process of norm selection.* Our paper addresses this interesting issue, studying a setting with incompatible individual preferences, when it is not clear, which behavior would be established as a norm, or whether a norm will result at all.

We focus on the particularly interesting case of heterogeneous populations [14]–[16]. By means of computer simulations, we study conditions under which shared norms result in situations where heterogeneous, incompatible preferences prevail. Examples for this type of situation abound. One may think of groups that move into a different neighborhood or of the differentiation of behaviors, e.g. when young people grow up and develop disparate preferences. What happens in such situations? Under what conditions will the new behavior spread and prevail? When will multiple behavioral norms coexist in space? When will the population fail to develop any kind of norm in the sense that all individuals behave according to their own preferences? This is “perhaps one of the most fundamental problems that the social sciences have ever tackled” [17].

Models of social norms have been developed mainly from two different perspectives. On the one hand, game-theoretical models focus on individuals’ preferences for different behaviors and treat norms as a means to change others’ decisions in order to decrease free-riding and to facilitate coordination [9]. These models have helped identify critical conditions of norm emergence, which will be discussed below. However, game-theoretical models typically focus on individuals’ behavior in equilibrium and, thus, do not inform about the *processes* that lead to the development of these stable states. Models of social influence [18]–[21], on the other hand, typically abstract from these preferences (an exception is the models by [22]–[23] and focus on the social influence processes that underlie consensus or norm formation. One important advantage of this approach is that it makes explicit how influence between pairs of individuals can result in the emergence of shared behavioral norms or in behavioral diversity. Our core contribution to the literature is that we integrate these two perspectives, by modeling explicitly that individuals often have preferences for different behavior and by analyzing the dynamics resulting from social interactions. The integrative model allows studying the processes that give rise to social norms in populations where individuals have incompatible motives in the sense that they prefer different behavioral options. We use this model to identify, for example, conditions under which such populations develop shared social norms and conditions under which several norms can coexist.

To this end, we will present an agent-based simulation model of norm emergence. That is, we seek for a mechanism-based explanation of typical macro-level observations based on micro-level interactions of individuals [24]. Agent-based simulation models are one useful way to achieve theoretical progress: they “are used to perform highly abstract thought experiments that explore plausible mechanisms that underlie observed patterns” [25]. More specifically, they offer the possibility “of modeling individual heterogeneity, representing explicitly agents’ decision rules, and situating agents in a geographical or another type of space” [26].

The contribution of this paper can be summarized as follows. A new mechanism of norm emergence is proposed, which allows to understand macro-level observations as outcome of dynamical self-organization processes that results from micro-level assumptions. Our main question is how and when shared norms emerge in societies with groups that have incompatible preferences and no shared regulatory interest. The model is built on basic factors of norm emergence that have been discussed in the literature (externalities, interactions in social networks, and sanctioning efforts). However, our model goes beyond the existing theories as we study the dynamic interplay between these factors and heterogeneous preferences. To explore these non-trivial complex dynamical processes, we use computer simulations. These establish an explanatory relationship between the starting point and result of social processes, and they reveal counter-intuitive properties of norm formation: for example, the minority may adjust its behaviour to the preferences of the majority, or the minority may establish an unfavorable norm, or different behaviours may co-exist, depending on the respective history of the social system (i.e. the “initial conditions”).

There is no general agreement regarding how to define social norms [27]. We use here a minimal definition, which is often employed in the literature: norms refer to common behaviors. Many definitions include sanctioning efforts as a typical aspect of social norms. We treat sanctioning as a separate variable and examine how different kinds of sanctioning affect the behavioral macro-level outcome. In accordance with [9], we distinguish between coordination norms (“behavioral conventions”) and cooperation norms. Coordination norms are self-enforcing, i.e. they occur by themselves, as everybody profits from following them. A typical example is the convention of pedestrians to walk on one side [28]–[30]. Cooperation norms, in contrast, are *not* self-enforcing, i.e. they do not automatically emerge, because each individual has an incentive to deviate from the norm. Therefore, the establishment of cooperation norms has often been considered to be a public goods or prisoner’s dilemma kind of problem: Cooperation is needed, but unlikely, because it requires that some or even all people overcome selfish behavior, at least temporarily. This theoretical idea is developed, for example, in the work of Demsetz and Coleman [31]–[32]. In our contribution, we will focus on situations where norms are not self-enforcing because individuals have heterogeneous, incompatible preferences, and where it is initially not clear what norm may emerge.

### Theories of Norms

The literature distinguishes two basic processes of establishing norms: norms can be intentionally created by human design and established by social control [33], or they can emerge spontaneously and informally [6], [34]–[36]. The implementation of laws is an example for the first process (a perspective often taken by legislators and, sometimes, political scientists), whereas the emergence of shared behaviors in peer groups exemplifies the second process (as typically studied in sociology). Here, we study the case where a normative entrepreneur does *not* exist and focus our analyses on *spontaneous, endogeneous* norm emergence.

Existing formal models of norm emergence have taken either a game-theoretical perspective or focussed on the social-influence processes that underlie norm emergence. Game-theoretical approaches are concerned with norms primarily in the context of coordination or cooperation problems. They are typically based on ultimatum games [10], [37]–[40]), stag hunt games [41], or prisoner’s dilemmas [9], [17], [42]–[43]. The prisoner’s dilemma approach is probably the most pertinent one, treating cooperation norms as public goods that are unlikely to occur without a cooperation-promoting mechanism such as repeated interations [42], [44]–[45], trust and reputation [46]–[48], signaling [49]–[57], punishment [32], [58]–[61], or social control [62]–[64].

The following three factors described in the game-theoretical literature on social norms are used to set up our explanatory model: externalities, frequent interactions in a social network, and sanctioning. *Externalities* exist when the behavior of an actor imposes costs or benefits on other actors and, thus, create incentives to influence others’ behavior. Thomas Hobbes [33], for example, argued that a war of all against all (a situation where all actors impose extensive externalities on others) would promote the creation of a state that enforces formalized social institutions in order to constrain individual behavior. Demsetz [31] points out that norms (he speaks of “property rights”) emerge if the costs of “internalization” of the externalities are lower than the gains. Here, “internalization” means that the costs of an externality are borne by those who cause them. However, when the costs of an externality are low and the costs of norm creation are high, then the benefits of the generated norm will be low. As a consequence, the norm will not emerge. Under this condition, there is no demand for a norm ([32]). But even if externality costs are large, the costs of internalization may still be too high.

The second factor that promotes norm formation is the *interaction in a social network*. For instance, Ellickson [11], argued that the formation and enforcement of cooperation norms is likely to happen “close-knit groups”. Ellickson’s example refers to the solution of conflicts about trespassing cattle causing various externalities. He argued that close relationships between the farmers promote the emergence of norms regarding conflict regulation: in such a community there is an interest in longstanding relationships, which creates incentives to compensate victims of externalities and thereby to reduce negative externalities. Likewise, Coleman [32] argued that the integration into social networks encourages sanctioning and, consequently, the emergence of norms.

The third relevant factor for the emergence of social norms, which is related with the second, is *sanctioning* (or punishment). Examples are the theories by Heckathorn [65]–[66], Coleman [32] and Ellickson [11]. Sanctioning may help to reduce externalities. However, whether sanctioning is imposed depends on the costs of sanctioning for the sanctioner as well as for the sanctionee.

There is a vast number of empirical studies about the effects of sanctioning, in particular in criminology [67] and in sociology of social movements [68]. In particular, experimental research shows that sanctions are imposed on defectors, actors who do not contribute to group goals [69]. In addition, individuals may sanction even if they are not directly affected by norm violations [70].

Social-influence models highlight another aspect of norm emergence. These models typically abstract from the fact that individuals may have opposing preferences and decide strategically to maximize their own benefit, which is the central aspect of game-theoretical approaches. Instead, social-influence models focus on the assumption that individuals are open to influence from others and adjust their behavior to increase the similarity with others [19]–[20], [62], [71]–[77].

Social-influence models typically assume that the behavior is uniformly distributed in the beginning of the norm formation process, a state without social order. Eventually, however, repeated social influence on the level of individuals decreases the variance of opinions in the population and leads to the emergence of a globally shared norm. In fact, one of the most classical insights from these theoretical models is that populations will, after a sufficiently long time, reach a state of perfect uniformity, if no subset of individuals is perfectly excluded from social influence [18], [78]. In tandem with homophily [79]–[80], the tendency to interact with similar others, social-influence can also lead to a coexistence of multiple norms [19]–[20], [71]. Modelers assume that individuals are not influenced by others that are perceived as being too dissimilar. Under this condition, the population can fall apart into separate subgroups, which develop different social norms.

## Methods

### A New Model of Norm Emergence

As science progresses, models must be able to capture an increasing body of theoretical insights and empirical evidence. However, it is often difficult to develop a model that is able to reproduce *many or even all* known facts and relevant features of previous models. The reproduction of multiple facts is a good indicator for the explanatory power of a model. Therefore, we list various stylized facts of social norms in the following. The goal and challenge of our study will be to develop a model that is consistent with all those facts, thereby offering a useful generalization of previous work on social norms:

1. The occurrence of social norms requires (positive or negative) externalities, such as material damages (like environmental destruction) or intangible effects (such as being dissimilar to others). Externalities make a coordinated behavior desirable [10], [29]–[30], [32], [65], [81]–[82].
2. The establishment and maintenance of norms requires punishment of norm deviation or rewarding compliance [34,58,60, 83–86.
3. Norms are most likely to emerge in close-knit groups, i.e. when there is a social network in which individuals frequently interact [11].
4. Norms may have almost *any* content, but it is largely history- or path-dependent [30], [87]–[89].
5. It is possible that unpopular norms are established, which are not aligned with the preferences of the majority [77], [90]–[91].
6. Typically, one finds a local consensus and global diversity, i.e. different local norms in different places [40], [71].
7. Norms can vary abruptly from one area to another and from one group to another. The separating borders are quite sharp (Switzerland’s “Röstigraben”, a cultural faultline, provides a well-known example; for a more general theoretical treatment, see also [15]).

The model proposed in this paper is covering all these aspects. While the first three points are directly implemented in our simulation model of norm formation through its micro-level assumptions (see this section and the one on The Multi-Population Norms Game with Punishment), the other points are not part of the model ingredients.

**Figure 1:**
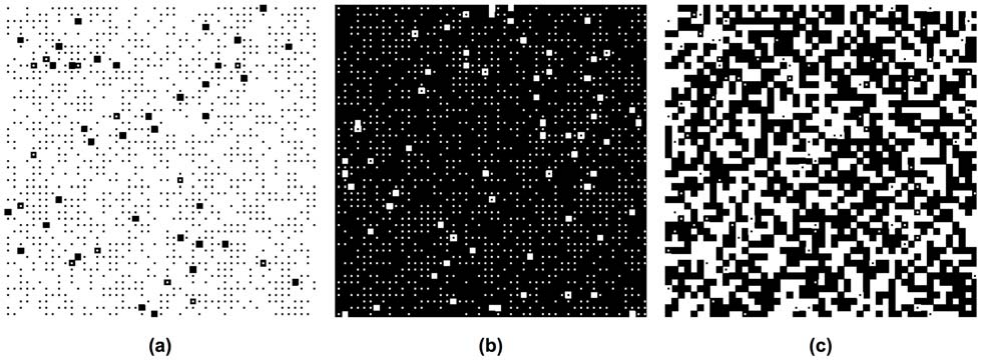
Path dependence in the establishment of social norms in case of two equally strong, interacting populations (*S* = 0.5). Both preferences are equally distributed in space. (a) If the initial average commitment to the preferred behavior is high in population 1 (*p*_1_ = 0.9), but small in population 2 (*p*_2_ = 0.4), the preference of population 1 establishes as commonly shared behavior, i.e. as social norm. Only a few individuals deviate, primarily due to random behavioral changes (determined by the rate *r* of strategy flips). (b) If the initial average commitment is high in population 2 (*p*_2_ = 0.9), but small in population 1 (*p*_1_ = 0.4), the preferred behavior of population 2 will set the norm. The baseline parameters of all simulations are *A* = 1, *B* = 0.5, R = 10, and *r = 0.01.* (c) If the initial average commitment is the same in both populations (*p*_1_ = 0.5, *p*_2_ = 0.5), members of both populations predominantly do what they prefer. Only a few individuals deviate from their preferences due to interaction effects or random behavioral changes (see squares with dots in the middle).

**Figure 2:**
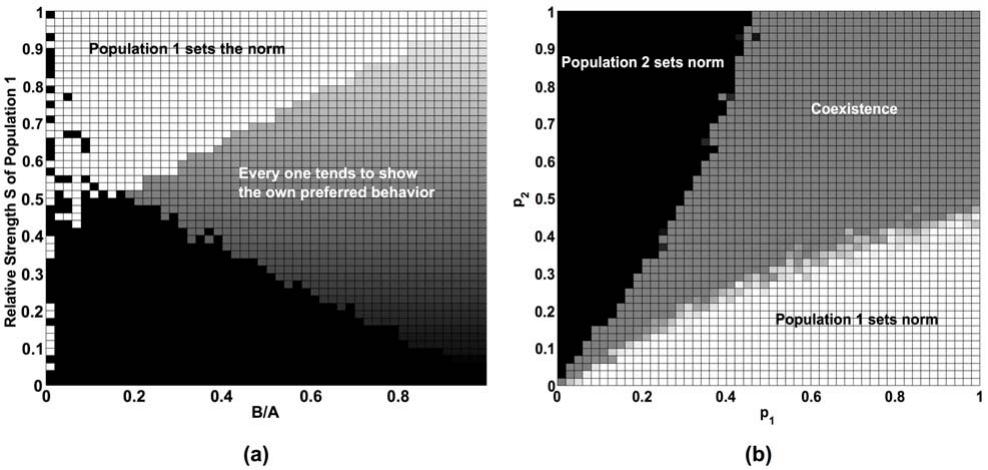
Phase diagrams illustrating how the finally resulting system behavior depends on the parameter choices and on the initial conditions. Left: Resulting outcome as a function of the relative benefit *B*/*A* of showing the preferred behavior and the relative strength *S* of population 1 for the model parameters *R* = 10, *r* = 0.01. Three different cases (“phases”) are possible, as indicated in the figure: either the great majority of people shows the individually preferred behavior (grey), or the behavior preferred by population 1 is established as a norm (white), or alternatively the behavior of preferred by population 2 (black). Right: Resulting outcome as a function of the initial average commitments *p*_1_ and *p*_2_ to the respectively preferred behavior in both populations for the model parameters *A* = 1, *B* = 0.5, R = 10, *r* = 0.01, and *S* = 0.5.

**Figure 3:**
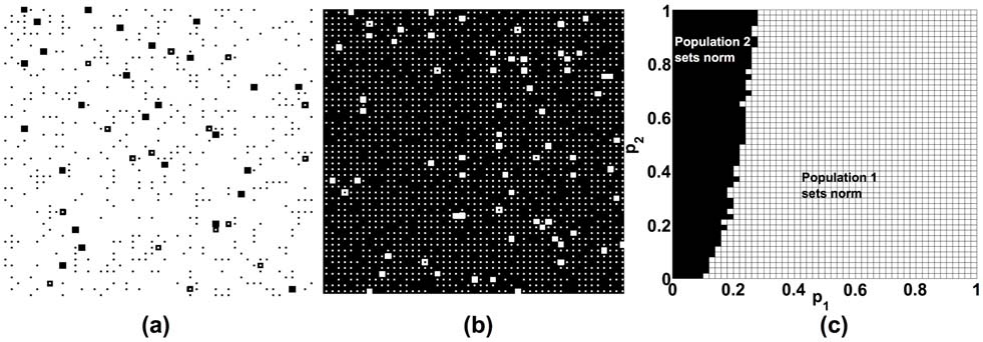
Due to the path dependence of norm formation, unpopular norms can be established, if the unpopular behavior is initially overrepresented. In our computer simulations, 80% of individuals belong to population 1 and prefer behavior 1 (*S* = 0.8). The other model parameters are *A* = 1, *B* = 0.5, *R* = 10, *r* = 0.01. (a) If the initial average commitment is the same in both populations (*p*_1_ = *p*_2_ = 0.5), the behavior preferred by the majority wins through, as expected. (b) However, if the initial average commitment in the minority population is high (p_2_ = 0.9), while it is low in the majority population (p_1_ = 0.25), the behavior preferred by the minority establishes as a norm. (c) The dependence of the final outcome on the initial average commitments p_1_ and p_2_ can be illustrated by a phase diagram. In this case, the results are for *A* = 1, *B* = 0.5, *R* = 10, *r* = 0.01, *S* = 0.8.

**Figure 4:**
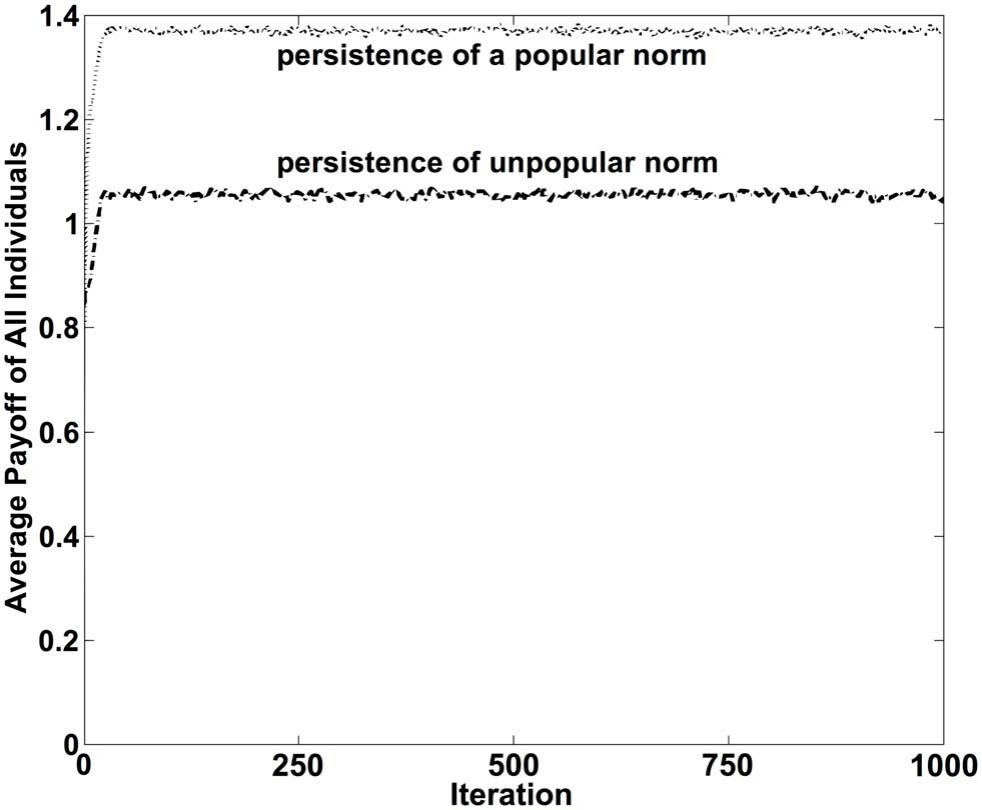
Average payoff of all individuals in the course of time t for the two scenarios illustrated in Fig. 3, assuming the parameters *A* = 1, *B* = 0.5, *R* = 10, *r* = 0.01, *S* = 0.8. Dotted curve on the top: the behavior preferred by the majority wins through (*p*_1_ = 0.5, *p*_2_ = 0.5), Dashed curve on the bottom: the behavior preferred by the minority establishes the norm (*p*_1_ = 0.5, *p*_2_ = 0.5). Note that, compared to the (initial) coexistence of the two behaviors, the average payoff increases even, if the behavior preferred by the minority wins through.

**Figure 5:**
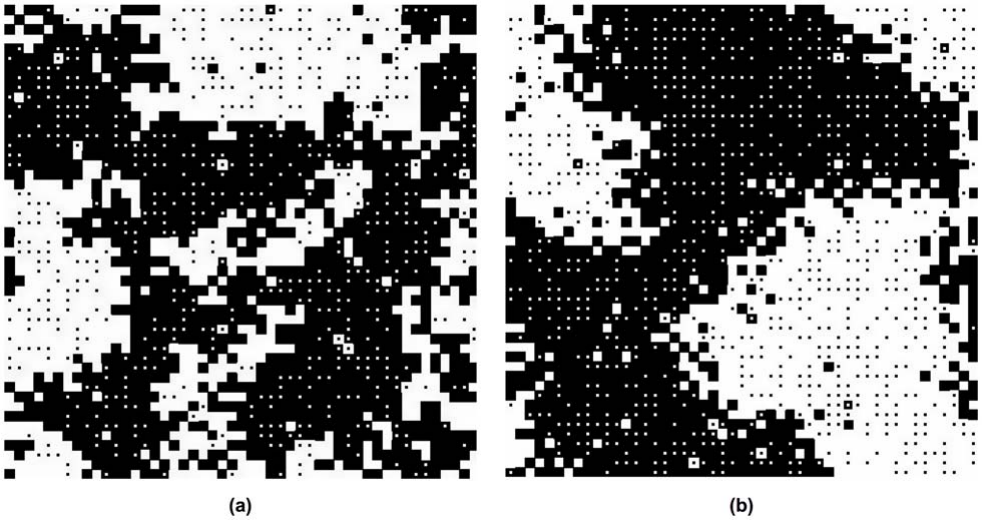
Emergence of local conformity and global diversity, when the interaction range *R* is small. The displayed snapshots are from computer simulations for two equally strong populations (*S* = 0.5) and taken after *t* = 100 iterations (a) for *R* = 1 and (b) for *R* = 2. The other model parameters are *A* = 1, *B* = 0.4, *S* = 0.5, *p*_1_ = 0.5, *p*_2_ = 0.5, *r* = 0.01.

**Figure 6:**
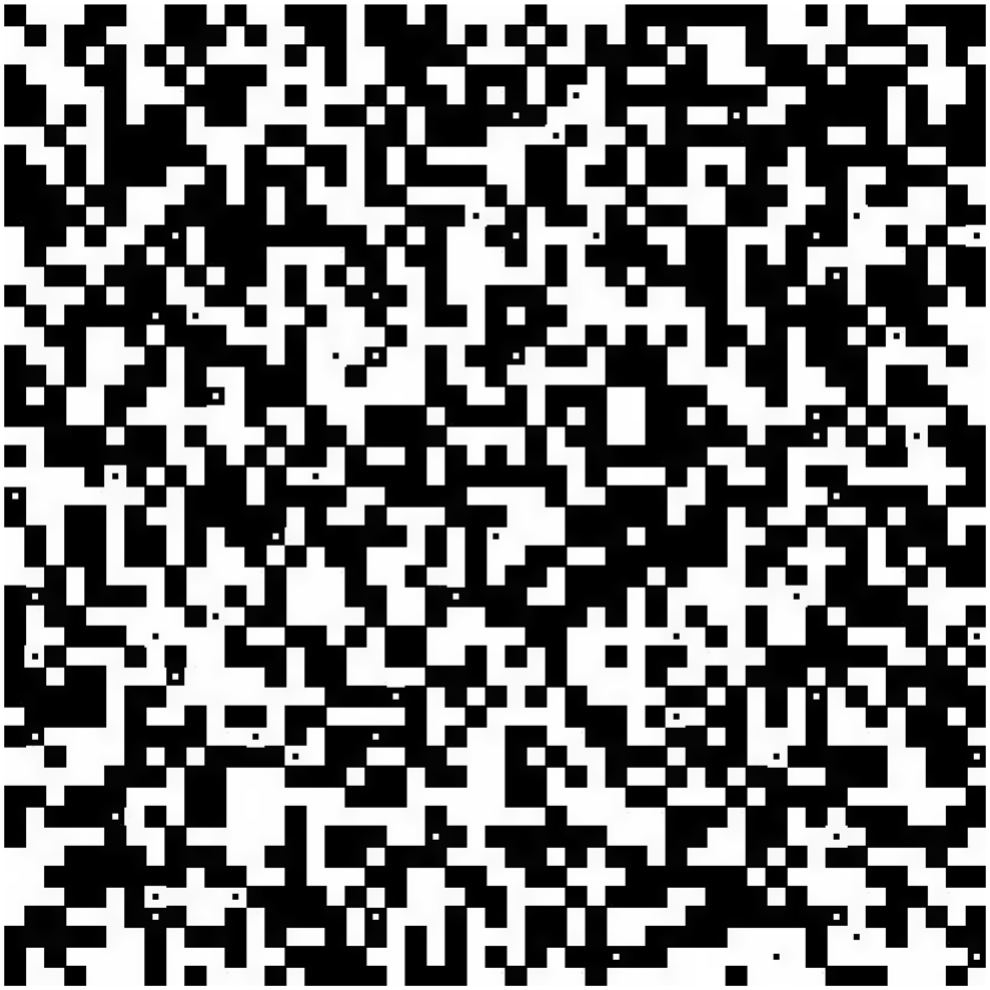
Coexistence of behaviors, when the benefit *B* of pursuing the individually preferred behavior is larger than the advantage *A* of conforming with the behavior of the respective interaction partner. The model parameters used in this computer simulation are *A* = 1, *B* = 1.2, *R* = 10, *S* = 0.5, *p*_1_ = *p*_2_ = 0.5, *r* = 0.01, i.e. the only parameter that is different from the ones used in Fig. 1(a) is *B*.

**Figure 7:**
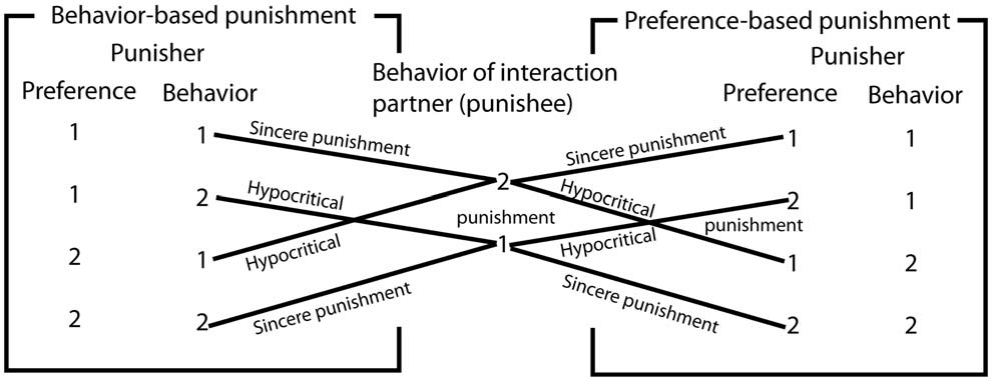
Illustration of our typology of four different kinds of punishment, distinguishing behavior-based (left) and preference-based punishment (right) on the one hand, and sincere and hypocritical punishment on the other hand.

**Figure 8:**
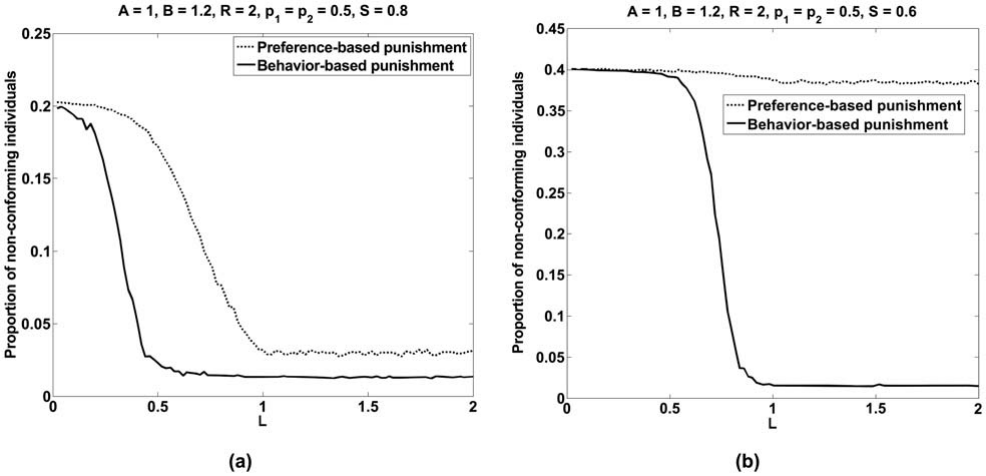
Proportion of individuals showing behavior 2 as a function of the punishment level *L*, when individuals either apply behavior-based or preference-based punishment. (a) For *S* = 0.8, preference-based punishment is less successful in establishing a commonly shared behavior 1 than behavior-based punishment. (b) Preference-based punishment may fail completely, if both populations have a similar strength (*S* = 0.6). The other model parameters are *A* = 1, *B* = 1.2, *R* = 2, *p*_1_ = 0.5, *p*_2_ = 0.5, *r* = 0.01.

**Figure 9:**
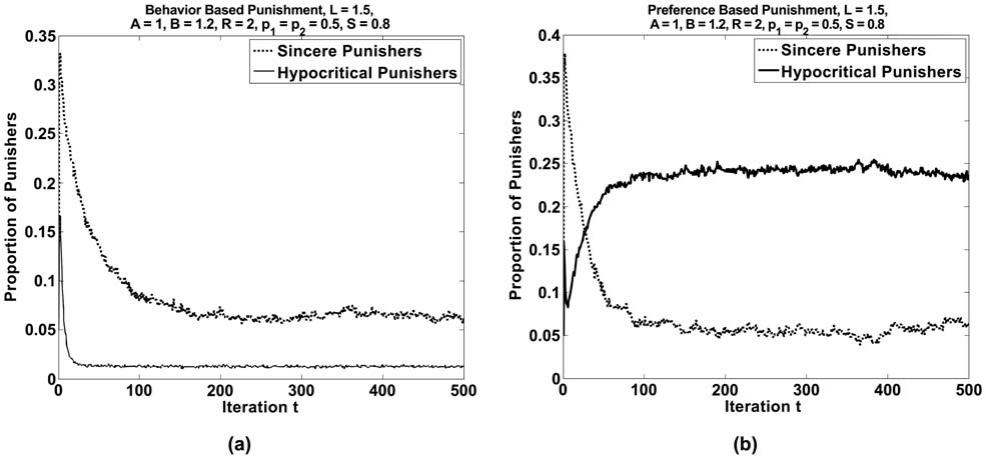
Proportions of sincere and hypocritical punishers as a function of time t in case of (a) behavior-based punishment and (b) preference-based punishment. The model parameters in both simulation scenarios are *A* = 1, *B* = 1.2, *L* = 1.5, *R* = 2, *r* = 0.01, *S* = 0.8, *p*_1_ = 0.5, *p*_2_ = 0.5.

**Figure 10:**
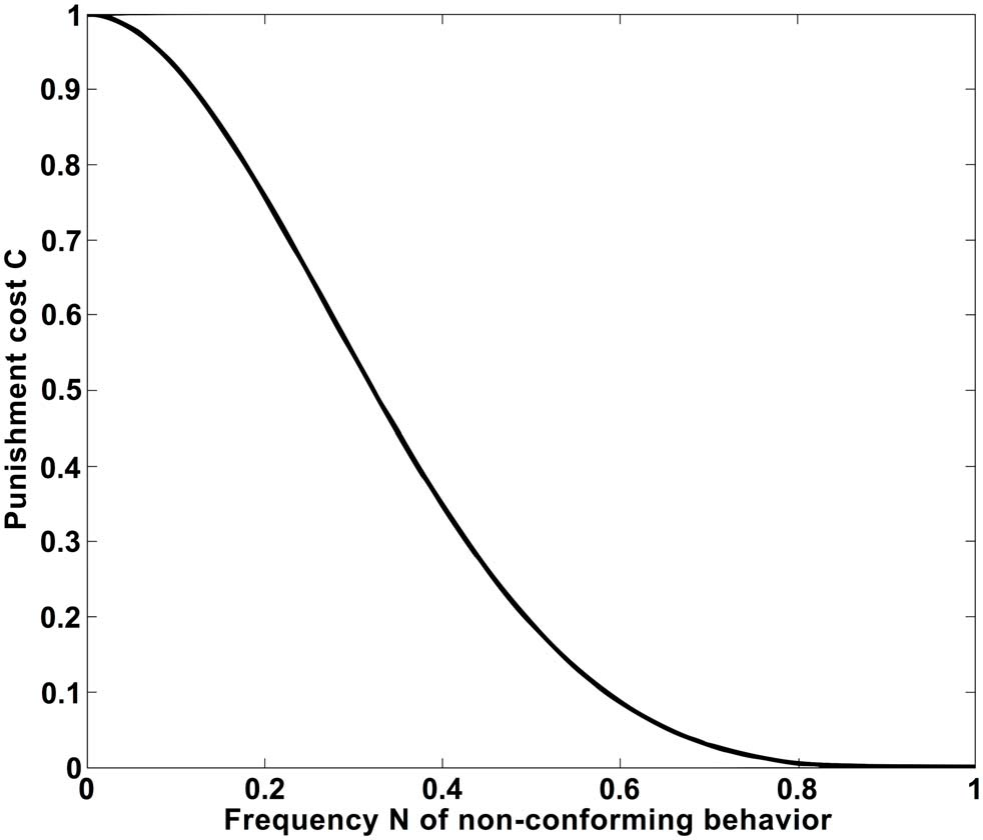
Adaptive group pressure is introduced in our multi-population norms game by specifying the punishment cost *C(N)* = *C*_0_ (1-*N*)^4^[2-(1-*N*)^2^]^2^ as a function of the relative frequency *N* of non-conforming behavior in the interaction neighborhood of range *R* (here: for *C*_0_ = 1).

**Figure 11:**
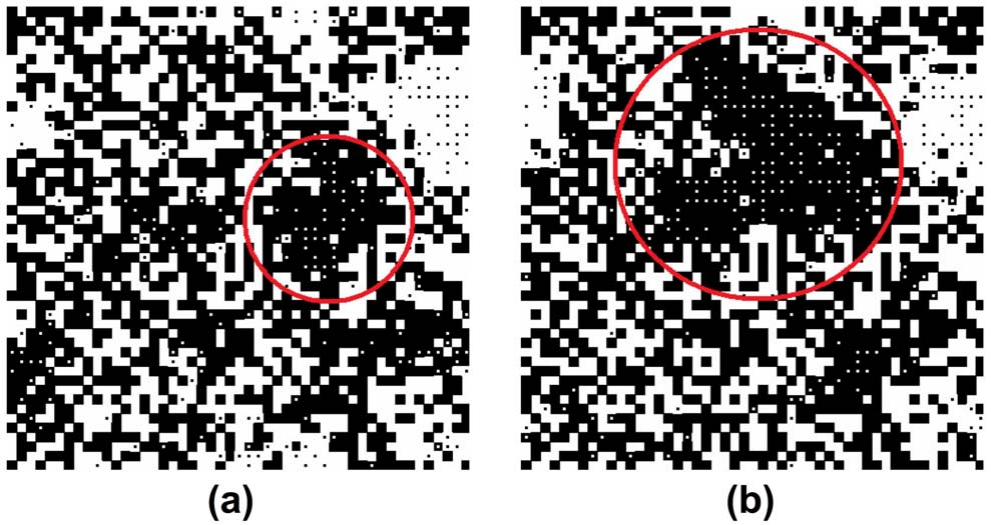
Adaptive group pressure causing the spontaneous birth and spreading of a social norm after many iterations: (a) *t* = 250, (b) *t* = 1000. The model parameters are *A* = 1, *B* = 1.1, *C*_0_ = 1, *R* = 2, *k* = 3,*r* = 0.01, *S* = 0.5, *p*_1_ = *p*_2_ = 1.

**Figure 12:**
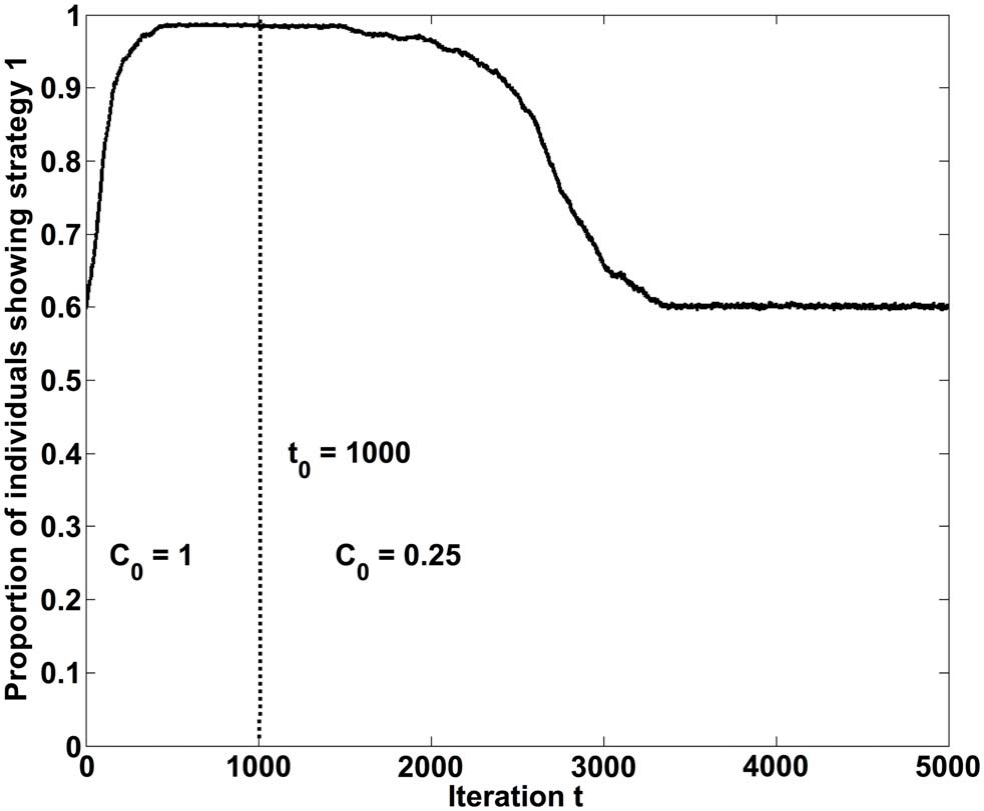
Persistence of a social norm, when sanctioning efforts are stopped at some point *t*_0_ in time. Before this time, behavior 1 has been established as social norm. The model parameters used in the computer simulation are *A* = 1, *B* = 1.1, *R* = 2, *r* = 0.01, *S* = 0.6, *p*_1_ = *p*_2_ = 1, *k* = 3 and *C*_0_ = 1 until *t*_0_ = 1000, afterwards *C*_0_ = 0.25.

We will demonstrate that they rather *result* from social interactions, i.e. they can be understood as emergent macro-phenomena within the framework of our evolutionary dynamical model. Figure 1 presents three different, typical simulation outcomes. Figures 2 and 3 show that any of the two preferred behaviors may be established as a norm (corresponding to property 4 above). Figure 4 demonstrates the possible existence of unpopular norms (property 5). Global consensus despite global diversity (property 6) is found in Figure 5. The same figure illustrates that norms may change abruptly from one place to another (property 7). Under conditions, for which individuals would usually just show their own preferred behavior without sanctioning efforts (Figure 6), Figures 7 to 9 demonstrate surprising effects of sincere and hypocritical punishment. Finally, Figures 10 to 12 show the interesting effect that adaptive group pressure (where randomly emerging local majorities punish local minorities) can establish norms, starting from local clusters [42. 92]. Remarkably, in our model, these are created by accident under conditions, where individually preferred behaviors would usually coexist.

In the following sections, we introduce the model ingredients step by step. We start with the effect of social influence, add the element of sanctioning (punishment), and then allow sanctioning to be adaptive (depending on the local majorities). To illustrate the relevance of each model ingredient, we discuss the resulting properties along with each new ingredient.

In our model, individuals are distributed on a checkerboard-like, two-dimensional spatial grid with periodic boundary conditions, i.e. individuals at the edge of the grid interact with individuals on the opposite side of it, as if the edges of the square grid would be connected to form a torus. Hence, everybody has the same number of neighbors. This space represents geographical space. In our simulations, the grid is fully occupied by so-called agents, which represent individuals. Agents do not relocate to other places, but they interact with other agents within a certain range *R*. Agents may show one of several behaviors *b*, and they have certain preferences *p*. These preferences are reflected by the payoffs *P_b_*. A high preference means a high payoff (intrinsic satisfaction), if an individual performs her preferred behavior. When a less preferred behavior is shown, the related payoff is lower, zero, or even negative.

We focus on the case of two alternative behaviors *b* only. Thus, some agents may prefer behavior *b* = 1, while the others prefer behavior *b* = 2. Hence, based on their respective preferences *p*, agents can be subdivided into two different (sub-)populations. Behavior and preference (or belief) do not have to be consonant with each other, i.e. individuals may show the behavior they prefer (*b = p*) or not, (*b≠p*). Showing the preferred behavior yields a payoff *P_b_ = B*>0, which reflects the *benefit* of doing what the individual prefers. Otherwise, the intrinsic payoff is assumed to be *P_b_ = 0*.

In addition, if two interaction partners show different behaviors *b* and *c*, there is no additional payoff. However, individuals are assumed to have an advantage *A*>0, when *conforming* with the behavior *c* of an interaction partner. This advantage could reflect an intrinsic satisfaction to interact with similar others.

Our computer simulations start with uniformly distributed preferences and perform a random sequential update in three substeps: (a) Interactions with a payoff, (b) imitation, and (c) randomization.

- **Interaction.** With probability 1/*N*, one of the *N* agents (“agent 1”) is randomly chosen as *focal agent*. Then, an *interaction partner* (“agent 2”) is chosen within a certain radius *R* around the location of this agent. The interaction between both agents generates a payoff *P_b_* for the focal agent and a payoff *P_c_* for the interaction partner, which depends on the behaviors *b* and *c* of both agents and their respective preferences *p* and *q* (where *q* represents the preference of the interaction partner). Table 1 shows the corresponding payoff matrix.
- **Imitation.** Afterwards, another interaction partner of agent 1 (“agent 3”) is randomly chosen within the radius *R*, namely among those agents who prefer the same behavior. If that interaction partner obtained a higher payoff during the last interaction, agent 1 imitates [93]–[96] the behavior of that interaction partner with a probability proportional to the payoff difference (where the proportionality factor is 1/(*A*+*B*) with the payoff values of *A* and *B* introduced below). According to the “proportional imitation rule” [28], [97], agent 1 otherwise sticks to the previous behavior. The same imitation step is applied to agent 2, but with a separately chosen interaction partner (“agent 4”).

**Table 1:** Payoffs of the focal individual in the multi-population norms game (*A* = advantage of conforming with the behavior of the interaction partner, *B* = benefit of showing the own preferred behavior).

		Behavior *b* of the focal individual conformswith the behavior *c* of the interaction partner	
		Yes	No
Focal individual performs preferred behavior *p*	Yes	*A+B*	*B*
	No	*A*	0

We restrict this imitation step to in-group interactions, which appears to be justified by homophily: individuals are more easily influenced by people who can serve as “role model”; in contrast, it would certainly make little sense to copy the behavior of somebody with different preferences, as this would often lead to disappointing results. One possible extension may be a specification, where imitation is allowed in larger groups and among agents who are not in spatial proximity. Such variations would model faster and less spatially dependent communication structures, capturing the trend towards online social media and the meanwhile widespread usage of smartphones. Possibly, such changes would result in qualitatively similar processes; however, with faster convergence. We leave such model variations to future studies and focus here, for the sake of a greater in-depth understanding, on the basic micro-macro dynamics of our simpler scenario.

Note that, in our model, private preferences can be inferred from public actions. As long as a norm has not been established yet, agents show their preferred behavior most of the time (as is confirmed by our computer simulations).

- **Randomization.** In order to take into account trial-and-error behavior, mistakes, or other factors contributing to randomness in decision-making processes, we assume that individuals turn to the *opposite* behavior with a small probability *r* (*“random strategy flipping”).* Such a probabilistic model specification avoids artifacts that may easily occur in deterministic models [98].

After updating payoffs and behaviors of agents 1 and 2, the computer program selects another focal individual (the new agent 1), performing the same update steps as described before. When *N* individuals have been updated (i.e. after updating *N*/2 focal agents and their interaction partners in the interaction step), the simulation time *t* is increased by 1. Individual preferences are assumed to be constant in our model, i.e. they are not changed over time.

The payoff *P_b_* of an individual depends on whether its behavior *b* is preferred (*b* = *p*) or not (*b*≠*p*) and whether it conforms with the behavior *c* of the interaction partner (*b* = *c*) or not (*b*≠*c*). Further model parameters besides the *advantage A* of conforming with the behavior *c* of the respective interaction partner and the *benefit B* of showing the own preferred behavior *p* are the interaction range *R* and the rate *r* of random strategy flipping. *S* is the share of individuals belonging to population 1 (having the preferred behavior *p* = 1), and *p*_1_ denotes the average initial commitment of them to their preferred behavior, i.e. the fraction of individuals in population 1 actually showing behavior *b* = 1 at time *t* = 0. Similarly, *p*_2_ is the average initial commitment of population 2 to their preferred behavior *p* = 2.

From a game-theoretic perspective, if *A*>*B*, the model reflects a battle of the sexes with interaction partners of the *other* population [99], while simultaneously a stag hunt game (or coordination game) is played with interaction partners of the *same* population [29], [41]. Note that the structure of a stag hunt game also results when a prisoner’s dilemma game comes along with repeated interactions, reputation effects, or costly punishment [43], [100].

Given the model’s payoff structure, one would expect that an individual will choose the preferred behavior if the benefit *B* of showing the preferred behavior is higher than the advantage *A* of conforming with the interaction partner (*B>A*). The interesting case is, therefore, characterized by *A>B*, where the payoff for conforming with somebody else is higher than the benefit of showing the preferred behavior. What will happen under such conditions, and what will be the effects of noise and spatial interactions? Will individuals end up showing the same behavior and, if yes, which one, or will they still show their own preferred behavior? We will see that all these variants are possible, and that the macro-level outcome depends on several factors. In any case, the formation of a commonly shared behavior always constitutes a *(normative) dilemma* in our model: a commonly shared behavior and social norm does not automatically result, as individuals with different preferences have an incentive to deviate from the norm whenever *B*>0.

Before we investigate the role of sanctioning, we study the resulting dynamics of the basic model described above. For the time being, we will therefore use the term “norm” in the sense of commonly shared behavior. The related model will be called the multi-population norms game.

## Results

### Multi-Population Norms Game

Our computer simulations assume that a share *S* of all *N* individuals prefers behavior 1 (i.e. *SN* individuals belong to population 1), while a fraction 1-*S* of individuals has a preference for behavior 2 (i.e. (1*-S*)*N* individuals belong to population 2). Parameter *S* may be considered to reflect the *relative strength* (here: size) of both populations. If *S* = 1/2, both populations are equally strong, while population 1 is stronger than population 2, if *S*>1/2. Of course, different strength could also result from other factors than population size, such as material resources (money, weapons, etc.), social capital (status, social influence, etc.), charisma or moral persuasion.

In our computer simulations, each site of the spatial grid is occupied by one individual. The two different preferences are distributed over the two-dimensional grid in a random and uniform way, i.e. individuals with different preferences are mixed. A proportion *p*_1_ of individuals belonging to population 1 starts with their preferred behavior and are represented by white squares, while a proportion 1-*p*_1_ starts with their non-preferred behavior and are represented by black squares with a white dot in it. Analogously, a proportion *p*_2_ of individuals belonging to population 2 starts with their preferred behavior and are represented by black squares, while a proportion 1-*p*_2_ starts with their non-preferred behavior and are represented by white squares with a black dot in it. Hence, the central dot reflects the *preferred* behavior and the color of the surrounding frame to the *actual* behavior. *p*_1_ may be called the *average initial commitment* of individuals of population 1 to their preferred behavior (and similar for population 2). Values of *p*_1_ or *p*_2_ smaller than 1 can reflect situations in which the populations are not fully committed to their preferred behaviors.

This can result from path dependencies, random strategy flips, or interactions with individuals pursuing different behaviors. (The initial commitment often depends on historical factors. Of course, not only the average commitment to a norm, but also its content can change over time, i.e. a norm can change its “character”. In our model, we assume that the character changes much slower than the commitment, so that changes of the content can be neglected.) For example, when a social norm is established, the proportion of individuals showing the preferred behavior goes to values close to 0 in one of the populations. Besides, studying cases with *p*_1_, *p*_2_<1 or *p*_1_≠*p*_2_ reveals particularly interesting kinds of system dynamics.

When a commonly shared behavior is formed in the course of time, the great majority of sites either shows white squares (if the behavior preferred by population 1 wins through) or black squares (if the behavior preferred by population 2 wins through). If completely black and white squares are mixed, it means that most individuals do what they prefer. This is a situation, which we will call a *coexistence* of different behaviors.

### Path dependence

Our first set of computer simulations assumes *B<A*, a large interaction range *R* = 10, equally strong populations (*S = *1*/*2), and different initial fractions of individuals showing their preferred behavior in the beginning. Our simulations reveal a variety of possible outcomes (see Fig. 1). Even when all model parameters are exactly the same, the final outcome can be very different, depending on the initial conditions: If a majority of individuals in population 1 shows their preferred behavior in the beginning (*p*_1_>1/2), but not so in population 2 (*p*_2_<1/2), most individuals end up with this behavior (see Figs. 1a and 2). However, if a majority of individuals in population 1 initially shows the behavior preferred by population 2 (*p*_1_<1/2) and the individuals of population 2 are very committed to their preferred behavior (*p*_2_>1/2), the individuals of population 1 will adjust to this behavior, i.e. population 1 will assimilate to population 2 (see Figs. 1b and 2). Finally, if the fraction of individuals initially showing their preferred behavior is about the same in both populations (*p*_1_≈*p*_2_), none of the behaviors will gain the majority. In this case, the majority of individuals ends up doing what they prefer (see Figs. 1c and 2). Note that this pattern of coexistence is also obtained when *B>A*.

The dependence on the initial condition can be illustrated by a so-called “phase diagram”: Figure 2 reflects the path dependence in the formation of social norms. It also shows that a commonly shared behavior is *not* formed, if both populations have similar strength (S≈1/2) and everybody starts with the preferred behavior (*p*_1_ = *p*_2_ = 1) or the average initial commitment in both populations is about the same (*p*_1_≈*p*_2_). This demonstrates that norms are not self-enforcing in our multi-population norms game without punishment if *B*>0, a somewhat surprising outcome, as we have assumed with *A>B* that the advantage *A* of conforming with the behavior of others is *larger* than the benefit *B* of showing the own preferred behavior.

The left panel of Fig. 2 can be understood by the following simple consideration. Let us assume that a focal individual shows the own preferred behavior and that it is surrounded by a share *s* of conforming individuals. In this case, the payoff of the focal individual will be *B*+*sA*, while it would be (1-*s*)*A* when switching to the not preferred behavior (as 1-*s* is the share of individuals who would then conform with the behavior of the focal individual). A rational individual would, therefore, switch if *B*+*sA*<(1-*s*)*A*, i.e. if *B*/*A*<(1-2*s*). For *s* = *S*, this defines the separating line in Fig. 2a between the grey area of coexistence and the black area, where population 2 sets the norm.

### Relevance of relative strength and unfavorable norms

If one population is stronger than the other, one would expect that the preferred behavior of the stronger population would win (assuming *B<A* and R = 10, as before). Figure 3 shows simulation results for *S* = 0.8, i.e. 80% of all individuals belong to population 1. In fact, Fig. 3a demonstrates that most individuals of population 2 eventually adapt to the preferred behavior of population 1. This is, however, not the case for *S* = 0.6, i.e. when population 1 is only slightly stronger than population 2. Moreover, it can even happen that the behavior preferred by the weaker population 2 finally prevails (see Fig. 3b). For this, the proportion of individuals who initially show their preferred behavior must be significantly higher in population 2 than in population 1 (i.e. *p*_2_>>*p*_1_, see Fig. 3c). In other words, the minority may succeed in establishing their preferred behavior as a commonly shared behavior, if they are more committed to it in the beginning than the majority. This offers an explanation why unfavorable norms, i.e. norms that are not aligned with the preferences of the majority of people, are possible. We will come back to this interesting case below.

The possible dominance of a behavior preferred by a minority also implies that the formation of a behavioral norm does not necessarily establish a system optimum, as pointed out by Elster ([101]; [102]). This is illustrated more clearly in Figure 4, and it shows that emergent norms are not necessarily beneficial, or at least not the most beneficial solution for a social or economic system. In fact, social norms can be very costly for a large fraction of people, as the example of female genital mutilation ([103]) suggest.

### Local cultures

So far, by selecting a large value of the interaction range *R*, we have assumed that every individual could interact with many other individual. In contrast, the following simulation results are for *R = *1 or *R = *2. In order to make our results comparable with Fig. 1, we again assume *A>B* and equally strong populations (*S = *1*/*2). As Fig. 5 illustrates, we find similar simulation results in this case as for models describing the formation of local cultures [71].

The phenomenon of global diversity despite of local conformity is an interesting puzzle in the social sciences [76], [104]–[105]. It is well-known from the spatial distribution of languages or dialects [106], or also from local preferences for certain kinds of food (“regional specialties”), traditions, or habits. It is remarkable here that we do not need a separate model to derive this observation. It results as a special case of our model for the formation of a behavioral norm, if the interaction range of individuals is small.

Note that the regions of uniform behavior are not stable. They are continuously changing, i.e. the boundaries between areas with different cultures (i.e. different majority behaviors) are moving over time. However, after very long time periods, the final outcome of our computer simulations could theoretically be a “monoculture”, where one norm (or set of norms) is shared by everybody.

The case of global diversity despite local conformity is to be distinguished from the previously discussed case of behavioral coexistence (“individualism”). Local cultures (or, more precisely, local norms) seem to form for small values of *B/A* (see Fig. 5), while coexistence appears to occur for large values of *B/A* (see Fig. 6). In the first case, a large number of individuals shows the non-preferred behavior, i.e. they assimilate to the local culture, while the predominating behavior changes from one region to another. In the case of coexistence, in contrast, most individuals show their own preferred behavior. (We would like to note, however, that the term “culture” is normally used in a wider sense, representing a whole set of norms and potentially other human artifacts as well. Nevertheless, a model that creates local norms can be easily extended to consider the interaction of a variety of independent or interrelated norms).

### A Typology of Punishment of Norm Violations

In many cases, the payoff for conforming with others does not exceed the benefit of following the preferred behavior (i.e. *A<B)*. Under these conditions, it is plausible that different behaviors coexist, an expectation that is confirmed by additional simulation analyses (Figure 6). In other words, this payoff structure motivates most people to do what they like so that coordination on one norm is unlikely. The state of behavioral co-existence, however, may disappear over time, if sanctioning strategies are applied.

In this paper, we analyze peer punishment, where agents bear costs to punish each other for deviant behavior. For centralized punishment, where punishment costs are pooled to exert formal punishment (i.e. by the police and the justice system), we refer to the work of [85]. The study of “pool punishment” may be an interesting extension of our model, in particular in cases where individuals have too little resources to perform a punishment themselves. In such situations, they may pool their limited resources to establish an efficient, centralized punishment. Such scenarios are evolutionary plausible, as shown by [85] for the case of punishing free riders in public goods games.

We distinguish two types of peer punishment: *behavior-based* and *preference-based* punishment. These two cases can be further subdivided into *sincere* and *hypocritical* punishment. Heckathorn [66] defines punishment of non-cooperative behavior as hypocritical, if own behavior is inconsistent with punishment actions (in this case: ego is non-cooperative and punishes non-cooperative behavior of alter). Analogously, we say that someone behaves hypocritical, when she sanctions somebody who actually conforms to her own preference or behavior.

- **Behavior-based punishment** assumes that individuals impose a punishment fine on an interaction partner, whose behavior differs from the own *behavior.*

**Sincere** behavior-based punishment describes the case, where the punisher does not prefer and does not show the punished behavior herself.
**Hypocritical** behavior-based punishment describes the case, where the punisher punishes a different behavior, although it actually corresponds to the privately preferred behavior. A typical example is that somebody, who does not dare to follow the own preferences, punishes somebody else for taking the liberty to do so.

There are numerous examples of hypocritical behavior-based punishment, some of which are discussed in [91]:

- *Unintelligible publications:* In certain fields of academia, unintelligible texts are popular because nobody wants the risk to be blamed for trivial science. Those who publicly question the unintelligible scientific work are punished by others who privately disapprove the work as well.
- *Review system and quality management:* The evaluation and review system in science may be subject to hypocritical punishment, as many may dislike it but only few oppose it in order to avoid the impression of trying to hide incompetence. Those who oppose it are publicly punished for questioning the review system. A similar thing may applyto the whole evaluation and quality management culture, which has been established in the past years in business, health care, administrations, schools, etc.
- *Sexual freedom and homophobia:* Most individuals dreaming of sexual freedom did not support it publicly or even opposed it before the “summer of love” [107]–[108]. Even though this is not an issue anymore in many countries, most people still want to avoid the risk of being blamed as homosexual. Thus, soccer fans, colleagues at work, or school kids with homosexual preferences often engage in jokes against homosexuals.
- *Bullying:* In school-classes, some pupils are targets of bullying and mobbing. Other pupils are afraid to be the next victim. Therefore, they may engage in mobbing other victims as well, although they do not have personal objections against them.

b. **Preference-based punishment** assumes that individuals impose a punishment fine on an interaction partner who behaved differently from the own *preference*.

**Sincere** preference-based punishment describes the case where a non-preferred behavior is punished.
**Hypocritical** preference-based punishment describes the case where the punisher sanctions someone for a behavior that does not comply with her own preferences, while actually performing the same behavior. One could describe the situation as one in which the punisher admonishes those who cannot suppress similar “weaknesses” as she shows herself.

There are also numerous examples for hypocritical preference-based punishment, some of which are discussed in [66]:

- Public punishment of personal weaknesses like promiscuity, smoking, drinking, use of illegal drugs, laziness, untidiness, unpunctuality, or forgetfulness, although the punisher actually has these weaknesses as well. Think of someone who regularly comes too late to meetings, but blames others if they dare to come later to her own meeting.
- A famous example are television evangelists who blame others for being homosexual or clients of prostitutes, but are later uncovered as being homosexuals or clients of prostitutes themselves.

### The Multi-Population Norms Game with Punishment

Our further simulations focus on the case, where the payoff *B* for following the preferred behavior exceeds the payoff *A* for conformity, in which both alternative behaviors would coexist without punishment (otherwise, norms could anyway be established). We will study two different scenarios, assuming that ego (agent 1) punishes alter (agent 2), either when alter shows a different behavior than ego (behavior-based punishment) or when she behaves against ego’s preference (preference-based punishment). In our model, punishment causes two different kinds of costs: There is a punishment cost *C*, which is deducted from the payoff of the punisher, and a punishment fine *F*, which is deducted from the payoff of the punished individual (punishee). The punishment cost for the punisher and the punishment fine for the punishee matter in so far as they influence the evolutionary dynamics of the model. In particular, a high punishment cost or fine can change the payoffs so much that they may lead to behavioral changes. In our simulations, punishment is not carried out based on a strict cost-benefit analysis, but rather based on the fact whether or not the respective interaction partner behaves contrary to ego’s behavior or preference. In this sense, our simulation simplifies punishment as an unconditional, normative act, comparable to the notion of the Kantian categorical imperative or the notion of unconditional normative behavior in the framing literature [109]. It also fits the literature on learning models with an unconditional punishment of norm violations [77], [91]. Furthermore, it is consistent with experimental results of [5], according to which sanctioning can be irrational in the sense that individuals may enforce norms against their own preferences or at unreasonably high punishment costs (larger than the resulting rewards; see also the work on altruistic punishment [69]). This “irrational over-sanctioning” is typically an effect of metanorms to punish deviant behavior, and further amplified by social relations. (It seems that deviant behavior per se is considered “harmful”, as it causes extra coordination costs or implies the likelihood of transaction failures [40]. In this context, we would also like to mention the observation of antisocial punishment [110].

In our computers simulations of norm formation under behavior-based or preference-based punishment, the punishment cost is assumed to be *C*, and the punishment fine *F* is assumed to increase with the punishment cost [111]. For simplicity, we assume a linear relationship *F = kC*. If the punishment effect *F* is larger than the punishment effort *C*, as is usually assumed, the proportionality factor *k* satisfies *k>*1. Moreover, we presuppose that agents do not punish, if their cumulative payoff would become negative.

If *P_b_* is the payoff for behavior *b* and the interaction partner is punished, the focal individual remains with a payoff of *P_b_-C*, and the payoff *P_c_* of the interaction partner is reduced to *P_c_*-*F*. Analytical calculations suggest that behavior-based punishment has a similar effect as an increase of the effectively experienced advantage *A* of conformity. Hence, we expect that, in principle, norms can always be established, if the punishment level *L* = (*k*+1)*C* is large enough. This is confirmed by our computer simulations.

Figure 8 shows simulation results for *A<B* and various values of the punishment level *L*, assuming that 80% of all individuals belong to population 1 (*S* = 0.8). Figure 8 illustrates clearly that most individuals follow their preferences, if the punishment level *L* is small. However, as *L* is increased, individuals of the weaker population (i.e. population 2) increasingly conform with the behavior preferred by population 1. For sufficiently large punishment levels *L*, the great majority of individuals share a common behavior. This demonstrates the formation of a social norm through punishment under conditions, for which everybody would show the personally preferred behavior without negative sanctioning. For high enough punishment levels, there are only a few individuals who show a norm-deviant behavior, and this is just because of the randomness assumed in our model.

It is interesting to see what results are found, when punishment is preference-based, i.e. everybody tries to enforce the own preferred behavior. Performing the same analysis as for behavior-based punishment, we find the results of Fig. 9. Surprisingly, preference-based punishment is much less effective than behavior-based punishment. Sometimes, it does not work at all (see Fig. 8b). This bears some similarity with the work on collective action failure [62], [112]. The fraction of individuals sharing a common behavior increases only very slowly with the punishment level *L* = (*k*+1)*C*. Therefore, behavior-based punishment is expected to be considerably more successful in establishing social norms. This is probably the reason why, despite the many benefits of diversity [113]–[116], group pressure towards conformity is surprisingly common in social systems [83], [117]–[119]. Interestingly, peer groups often care more about conformity in behavior than about the preferences of their members, and it seems that their norms can have almost any content.

The surprising findings of Figs. 8+9 can be understood as follows: Preference-based punishment (see dotted lines in Fig. 8) cannot establish a norm, when there is approximately a “balance of power” (see Fig. 8(b), where 60% of the individuals belong to population 1). Then, there are just too many individuals who punish those who deviate from their preferred behavior. Hence, preference-based punishment can establish a norm only, if one population has a clear majority as in Fig. 8(a), where population 1 has a share of 80%.

For behavior-based punishment (see solid lines in Fig. 8), the punishment costs *C* and fines *F* change the formula for switching from the preferred behavior to the non-preferred one from *B*+*sA*<(1-*s*)*A* to *B*<(1-2*s*) (*A+C+F*). In other words, punishment effectively increases the advantage of conforming. Hence, even a small difference in the population strengths allows the establishment of a norm. (Assuming *s* = *S*, we can see that the contribution *C*+*F* moves the separating line in Fig. 2 between the coexistence of preferred behaviors and the formation of a norm.).

To understand Fig. 9, we assume that 80% of individuals belong to population 1 and that the great majority of it shows their preferred behavior 1. Moreover, in population 2, punishment lets more and more individuals change to their non-preferred behavior 1 over time. In Figure 9(a), the proportions of sincere and hypocritical punishers are shown for behavior-based punishment. As expected, the frequency of behavior-based punishments goes down over time as behavioral conformity increases. Sincere punishment basically occurs when the few individuals that still show behavior 2 are punished by individuals of population 1. Hypocritical punishment, in contrast, disappears almost completely. It occurs when the few remaining individuals who still show behavior 2 are punished by individuals of population 2 who switched to behavior 1. In conclusion, the final ratio between sincere and hypocritical punishment should correspond to *S*:(1-*S*), i.e. to the ratio between the strengths of both populations, which fits the numerical values well.

In Fig. 9(b) for preference-based punishment, the proportion of sincere punishers is similar to the previous scenario. It is again mainly related to the majority of individuals in population 1 punishing individuals in population 2 who show behavior 2. However, the proportion of hypocritical punishers is *much* higher than in the case of behavior-based punishment. This is because all people belonging to population 2 who have turned to behavior 1, hypocritically punish all those who show their non-preferred behavior 1. The frequency of this is expected to be about (1-*S*)*100%. Considering the random strategy flipping in our computer simulations, the level of hypocritical punishment is again well consistent with this theoretical expectation.

In conclusion, while behavior-based punishment stops after convergence, preference-based punishment is still performed by individuals who have turned away from their preferred behavior. While this might be seen as paradoxical, since this results in a punishment of people sharing the same preferences, it might be seen as an attempt to continue “fighting” for the own interests.

### Multi-Population Norms Game with Adaptive Group Pressure

We will now discuss the case where the punishment level *L* in the multi-population norms game with sanctioning is not a constant, but a variable. It seems natural to assume that it might depend on the relative frequency *N* of non-conforming behavior within the interaction range *R*. If the frequency of non-conforming behavior is high, individuals would normally not dare to punish a behavior that deviates from the own one. However, if the frequency of non-conforming behavior is small, individuals would be encouraged to punish non-conforming behavior of an interaction partner. This could, for example, be expressed by the following formula: *C*(*N*) = *C*_0_ (1-*N*)^4^[2-(1-*N*)^2^]^2^, where *C*_0_ can be interpreted as maximum punishment level (or intensity of the metanorm demanding to punish deviant behavior). The function *C*(*N*) is illustrated by Figure 10 and supported by findings in social psychology, according to which social pressure increases with the level of conformity (see in particular the classical studies by [120]). The chosen functional form assumes low punishment levels when the non-conforming behavior is in the majority (*N*>1/2). However, when the conforming behavior prevails within the interaction range *R* (*N<*<1/2), the punishment level is high. According to this specification, individuals are assumed to always have a tendency to apply a certain level of punishment in favor of conformity. However, the conformity pressure exerted by someone is assumed to be high only, if her behavior prevails in the interaction neighborhood (i.e. *N<*<1/2).

Figure 11 shows snapshots of a related computer simulation at different times *t*. The simulation studies the interesting case where most individuals would show their preferred behavior *without* punishment efforts (*A<B* and *C* = 0 = *F*). Moreover, we assume the maximum punishment level *C*_0_ to be high enough to reinforce a social norm when *everybody* initially shows the same behavior, but small enough to not support norm formation when different behaviors are uniformly distributed, as assumed in the beginning of our computer simulation (*t* = 0). Hence, most individuals show their preferred behavior for a long time period. Then, however, we discover a very surprising change in the system behavior. In some areas, local norms are suddenly showing up. These local norms eventually spread in the system. Eventually, global diversity disappears. Thus, under conditions where different behaviors would usually coexist, sufficiently strong group pressure will sooner or later establish a norm that is shared by a great majority and the resulting norm might have any content.

How can we explain the mechanism underlying this social dynamics? Due to a certain degree of randomness in the selection of individual behaviors (and interaction partners), it is possible that one of the behaviors gains a slight majority in a certain local neighborhood. Therefore, the local “balance of power” between the two competing behaviors is broken, when a sufficient number of “strategy flips” occur at the same time and creates a random bias for one of the behaviors. In order to cause significant deviations from the mean value, fluctuations in the individual behaviors have to randomly add up. Since deviations of significant size are rare, it requires a considerable time for them to occur by coincidence. However, once a sufficiently large deviation has appeared, the local majority increases its punishment efforts. According to Fig. 10, the group pressure is intensified to support the behavior of the local majority. As a consequence, the fraction of individuals showing the same behavior further increases. Therefore, when the local level of conformity is high enough, i.e. when a “super-critical cluster” of similar behaviors has occurred, that behavior can spread to neighboring areas. The need of a certain “critical” level of local conformity to trigger the formation and spreading of a norm establishes a threshold effect.

Once a super-critical (i.e. large enough) cluster has occurred, areas following the same norm are growing. The final competition between areas with different norms depends on the local majorities and on random events. Assuming equally strong populations, behavior 1 finally establishes the norm throughout the whole system with a probability of 50%, and behavior 2 wins through in the other 50% of cases, but this process may take a long time. For the parameter values assumed by us, there will always be a shared behavioral norm in the end. In our model with adaptive group pressure, individuals are only expected to stick to their personally preferred behavior forever, if the maximum punishment level *C*_0_ is small (i.e. the tolerance of other behavior is high).

The above explained threshold dynamics is fundamental to understand, why and how social order can be born spontaneously under conditions, where it would *not* occur in the absence of randomness. The restriction of social interactions to a sufficiently small neighborhood is crucial (i.e. spontaneous norm emergence is not possible for large values of the interaction range *R*, as coincidental majorities could hardly occur).

How stunning this finding is becomes more obvious by pointing out that spontaneous norm emergence would neither be possible for interactions of everybody with everyone else nor for randomly chosen interaction partners from arbitrary locations (i.e. inside and outside any radius *R*). The phenomenon is, therefore, not analytically comprehensible with a representative agent model. It requires the local co-evolution of individual behavior and social neighborhood to facilitate the process. Non-linear interactions, social proximity (in geographical or social space), time-dependence, and “noise” are ingredients absolutely needed for it. This stresses, in particular, the significance of randomness for an understanding of self-organization phenomena in social systems (otherwise no local behavioral majorities could emerge by coincidence).

### Persistence of established norms

The discussion above suggests that, for social interactions with adaptive group pressure, we would always find the formation of a social norm, whatever its content may be (if only *C*_0_ is high enough). Once a certain behavior has gained the majority, it would be reinforced and maintained over extremely long time periods. This history dependence might be interpreted as “hysteresis effect”. As a consequence, a social system would normally not switch from one norm to another, even if a norm is “outdated” and does not serve a purpose anymore (if it ever did). (According to [121], this phenomenon might be related to the development and subsequent maintenance of status value.) The only way of overcoming a once established norm in our model is the reduction of the maximum punishment level *C*_0_. Figure 12 illustrates such a scenario. It can be seen that an established norm persists over a long time, even if the punishment efforts are suddenly reduced. Despite setting the punishment strength *C*_0_ to a small value at some point *t*_0_ in time, we find a rather gradual decay of the support for the previous norm. The individually preferred behaviors are shown only after a surprisingly long transient state. Of course, an outdated norm may also be gradually transformed into another one, but this is outside of the scope of our model. In our model, the persistence of social norms is a consequence of the norm-creating mechanism. It does not necessarily require an internalization of the *content* of a norm [122], but rather the internalization of “normative thinking”, i.e. a metanorm of applying group pressure against behavior that deviates from the behavior of the majority.

### Sensitivity Analysis

In the following, we will discuss the sensitivity of our model with respect to parameter choices and model assumptions. The robustness with regard to parameter changes largely follows from the fact that the multi-population norms game *without* punishment can be analytically understood. We can conclude this from the circumstance that Fig. 2 (which studies how the macroscopic system behavior depends on the initial conditions, the relative strength of both populations, and the payoff values) is well consistent with findings in [123] (see Figures 3B and 4). Even though that paper is not focused on the particular kinds of social interactions studied here, it offers a mathematical framework that is general enough to cover our model. The correspondence of results holds despite the fact that no spatial interactions and no noise terms were considered in that paper. Apparently, a representative agent model describes the outcomes of the norms model without punishment well, and small noise does not change the results significantly.

A comprehensive analysis of the representative agent discussed here shows that there are no other system behaviors besides the ones discussed in that paper, and Figure 2 represents the possible cases and parameter dependencies well. The analysis of that paper further allows one to derive a number of generalized conclusions. For example, there are basically two parameters that matter: *A*/*B* and *S*. Moreover, the mathematical formulas presented in that paper can be applied to cases, where the parameters *A* and *B* are different in both populations, or where the two populations play other kinds of games. The formulas can also be extended to consider costly punishment. Effectively, the punishment costs *C* and punishment fines *F* = *kC* shift the experienced payoffs. For behavior-based punishment, for example, the experienced advantage *A* of conformity is increased by an average value of *F+C = *(*k*+1)*C*.

The applicability of the representative agent model ends, however, when adaptive group pressure is considered, which introduces additional non-linearity in our model of social interactions. As shown in Fig. 11, spatial interactions and noise make a pronounced difference in this case. This complicates matters so much that an analytical treatment becomes pretty much hopeless. In case of adaptive group pressure (see Fig. 10), the system can show “metastable” behavior, i.e. local fluctuations can create a systemic shift from one area of the parameter space to another area, which is a precondition for the spontaneous emergence of social norms. (This holds in particular when starting in the parameter area that supports a coexistence of different behaviors, see the grey parameter areas in the phase diagrams of Fig. 2). Despite the lack of analytical tractability, the macroscopic system behavior can still be intuitively understood with the information presented in Fig. 2. One must just consider that the phase diagram applies locally within homogeneous local clusters, and that (according to the threshold dynamics revealed by our simulations) behaviorally homogeneous clusters tend to grow when exceeding a certain size, while smaller clusters tend to shrink. Hence, the sensitivity analysis for the model *without* group pressure still gives a good intuition of what may happen in the system *with* group pressure.

There are just two additional parameters that matter: The noise strength and the range of interaction. In the limit of a large interaction range, the model implies the applicability of a representative agent model, which does not imply a spontaneous emergence of norms, if the benefit *B* of showing the own preferred behavior is larger than the advantage *A* of conforming with the behavior of an interaction partner. Under such conditions, the birth of a social norm is only possible when group pressure is imposed on minorities and the interaction range is sufficiently small. Furthermore, norms cannot spontaneously form, if the noise strength is zero. In such cases, the coincidental occurrence of local majorities is just not possible. However, realistic noise strengths will sooner or later lead to spontaneous norm formation. On average, it will take longer, when the noise level is small. In the limit of large noise strength, individuals will behave randomly: Local majorities may occur quickly, but they will also be quickly destroyed agan.

Finally, in-group imitation *is* relevant, as individuals would otherwise copy behaviors that do not make sense, considering their preferences. However, this assumption seems sufficiently justified by the empirical observation of homophily. As we have pointed out, private preferences can be inferred from public behaviors in the initial stage of the social dynamics.

## Discussion and Outlook

The problem of norm formation has been formalized as an issue of establishing cooperation with respect to a behavioral rule, analogously to a public goods or prisoner’s dilemma game. In contrast, we have proposed here a model of norm formation in situations where people have incompatible preferences. In our model, individuals tend to do what they prefer, but rewards or punishment may encourage conformity with the behavior of others.

Due to the incompatibility of preferences, several system behaviors are possible. Interestingly, even when each of the behaviors is preferred by the same number of individuals, interaction effects may cause behavioral consensus in favor of one behavior or the other, which corresponds to the spontaneous establishment of a commonly shared norm. However, for given model parameters, other kinds of outcomes may result, including the *coexistence* of different behaviors. This reflects the path dependence of the social dynamics in our computer model, and fits the empirical observation that social norms are history-dependent. Path dependence is a robust feature of social norms and has even been demonstrated after extreme events, such as earthquakes [88]. Therefore, the resulting path dependence from our model gives further plausibility of our simulation scenarios.

Furthermore, we compared two punishment scenarios. In the first scenario, individuals punish others for behaviors that do not conform with the own *displayed* behavior (behavior-based punishment), while in the second scenario, behaviors are punished that do not conform with the own *preferred* behavior (preference-based punishment). While the former could promote the formation of social norms, preference-based punishment seems to be effective in establishing a commonly shared norm. This implies that behavior-based consensus is harder to reach than value-based consensus.

When adaptive group pressure is considered, local social norms may spontaneously be created by random variations in individual behaviors, even in settings, where everybody would tend to show the personally preferred behavior. A precondition is that social interactions are focused on a sufficiently small neighborhood. Then, local behavioral majorities will sooner or later occur by coincidence, and group pressure will reinforce them. This gives rise to local conformity, i.e. local norms, which are then spreading into areas where everybody is still showing the personally preferred behavior. After a long period of global diversity, the system would finally end up with a globally shared norm. However, as the process of global convergence is very slow, it might happen that the content of the norm is changing locally, thereby creating new variants of a norm, before global consensus is actually reached.

A central advantage of our model is its simplicity. It allows studying the effects of punishment rules, the conditions of norm formation, the dependencies between relevant variables and parameters, and the dynamical patterns resulting in different scenarios. Despite the simplicity of our model, it can capture many characteristics of norm formation, including path-dependence, the possibility of unpopular or dysfunctional norms, the spontaneous occurrence and spreading of local cultures, or their long-term persistence when the punishment of non-conforming behaviors is reduced. The model therefore helps to get a better understanding of the mechanisms and dynamical processes underlying the formation of social norms, particularly when individuals have non-compatible preferences.

Our results are interesting also from a more general perspective. The identification of phase diagrams that demonstrate the existence of several possible outcomes (depending on the respective parameter values and previous state of the system) allows one to understand seemingly inconsistent empirical evidence with a unifying model. In fact, with one single model, we have been able to explain situations where no social norm is formed, where the majority sets the norm, or where unfavorable norms are established by a minority. While the study of phase diagrams is not entirely new, it can potentially offer a way of integrating several middle range theories into a unifying framework (see also [85] for an extensive discussion of phase diagrams for studying the emergence of cooperation). As such an approach might open the door for the formulation of (more) general theories in various areas of sociology, we consider it promising and propose to pay more attention to it.
